# ADAM-12 as a Diagnostic Marker for the Proliferation, Migration and Invasion in Patients with Small Cell Lung Cancer

**DOI:** 10.1371/journal.pone.0085936

**Published:** 2014-01-21

**Authors:** Shuhong Shao, Zunling Li, Wei Gao, Guohua Yu, Dexiang Liu, Fang Pan

**Affiliations:** 1 Institute of Medical Psychology, Shandong University School of Medicine, Jinan, P.R. China; 2 Department of Medical Psychology, Binzhou Medical Universtiy, Yantai, P.R. China; 3 Department of Biochemistry and Molecular Biology, Binzhou Medical Universtiy, Yantai, P.R. China; 4 Department of Pathology, Jinan Central Hospital, Jinan, Shandong, P.R. China; 5 Department of Pathology, Yuhuangding Hospital, Yantai, Shandong, P.R. China; National Taiwan University, Taiwan

## Abstract

Small cell lung cancer (SCLC) is highly aggressive and is characterized by malignant metastasis. Approximately 90% of patients die due to extensive metastasis. The extracellular matrix (ECM) is a natural barrier that can prevent cellular invasion and metastasis. Therefore, degradation of the ECM must take place in order for extensive metastasis to occur. A disintegrin and metalloprotease (ADAM) is a multi-domain protease that plays an important role in tumorigenesis, as well as tumor development, invasion and metastasis. However, there have been few reports on the expression and role of ADAMs in SCLC. In the current study, the expression and role of ADAMs in SCLC proliferation, invasion and metastasis was investigated. A total of 150 SCLC tissue samples were examined by immunohistochemistry for ADAMs expression. ADAM-12 was found to be abundantly expressed in 72.67% samples and other ADAMs were found to be expressed in 10% to 40% of samples. ADAM-12 levels in serum and urine, from 70 SCLC patients and 40 normal controls, were also measured using ELISA. ADAM-12 expression was significantly higher in SCLC patients than in healthy controls and in patients with extensive disease compared to those with more limited disease. Silencing the expression of ADAM-12 in H1688 cells through the use of specific siRNA significantly reduced cellular proliferation, invasion and metastasis. Supplementing the expression of ADAM-12-L or -S in H345 cells, significantly enhanced cellular proliferation, invasion and metastasis. Animal models with metastatic SCLC also exhibited increased expression of ADAM-12 along with enhanced invasion and metastasis. In brief, ADAM-12 is an independent prognostic factor and diagnostic marker, and is involved in the proliferation, invasion and metastasis of SCLC.

## Introduction

Small cell lung cancer (SCLC) is the most malignant of all lung cancers. The five-year survival rate is only 3–8% due to the widespread metastasis during the early stage and relapses that occur when resistance to the treatments develops [1]. Previously, the degradation of the extracellular matrix (ECM) has been the main focus of studies on the invasion and metastasis of SCLC [2], [3]. Matrix metalloproteinases (MMPs) have been detected in SCLC, and high expression levels of MMP-11 and -14 have been identified as independent negative prognostic factors in SCLC [4]. Inhibitors of MMPs have been used clinically for SCLC patients, but proved to be ineffective and did not improve the five-year survival rate of the patients [5]. This suggests that the degradation of ECM is a complex process and that proteases, other than MMPs, should be studied to determine whether other factors play a role in SCLC.

A disintegrin and metalloprotease (ADAM) belongs to the protease family. ADAMs can degrade ECM and shed the membrane-bound precursors that modulate cell-cell and cell-matrix interactions [6]. ADAMs are divided into two groups: membrane-anchored ADAM and secreted type ADAM. Secreted type ADAM contains thrombospondin motifs and is also called A Disintegrin and Metalloprotease with Thrombospondin Motifs (ADAMTS). ADAMs and ADAMTS can degrade ECM and shed precursors, thus promoting invasion and metastasis. Increased expression of ADAMs and ADAMTS has been detected in numerous tumors. ADAM-8, -12, -15 and -28 are highly expressed in non-small cell lung cancer [7], [8], [9], [10], ADAM-9, -12, -17 and -23 are highly expressed in breast cancer [11], [12], [13], [14] and ADAM-9, -12 and -17 are highly expressed in liver cancer [15], [16], [17]. ADAMTS4 and ADAMTS5 have been reported to be involved in the metastatic process by cleaving brevican in glioblastomas [18]. Interestingly, full-length ADAMTS1 was found to promote invasion and metastasis by shedding heparin-binding epidermal growth factor (HB-EGF) and amphiregulin, however the ADAMTS1 fragment displayed an anti-metastatic function [19]. SCLC is a strongly aggressive tumor. Approximately 90% of patients die as a result of extensive metastasis. Therefore, it is essential that a diagnostic marker be identified, and a prognostic factor be assessed for SCLC patients. Presently, there is no effective diagnostic marker for SCLC. There have been few studies examining the role of ADAMs expression in SCLC, with the exception of ADAM-15 [8]. Based on the potential significance of the role of ADAMs in promoting proliferation, metastasis and angiogenesis, we aimed to assess the expression levels of ADAMs and their relationship to clinical prognosis in SCLC in order to identify an effective diagnostic marker.

In present study, we found that the expression of ADAM-12 was higher in SCLC than other ADAMs via immunohistochemistry (IHC). Univariate and multivariate survival analysis indicated that ADAM-12 was an independent prognostic factor for SCLC patients. The expression level of ADAM-12 in serum and urine was higher in SCLC patients compared with healthy controls, as well as in patients with extensive disease compared to those with limited disease. Animal models demonstrating high metastasis of SCLC also had increased expression of ADAM-12 and enhanced invasion and metastasis. Our results supported the conclusion that highly presented ADAM-12 as an independent prognosis factor and diagnostic marker was involved in proliferation, invasion and metastasis in patients with SCLC.

## Materials and Methods

### Patients and cell lines

Formalin-fixed tumor tissue samples from 150 patients suffering from SCLC were obtained from Binzhou Medical University Hospital, China, between January 2008 and December 2011. Of the tissue specimens, 140 were obtained via biopsy and 10 were obtained during surgery. Basic patient information was obtained from patient files and is shown in **Table 1**.

**Table 1:** Patient characteristics and relative factors in 150 SCLC patients (Univariate Survival Analyses; log-rank test).

Characteristics		Patients (%)	Media survival (Mouth)	*P* Value
Age	<60	85 (56.67)	13.00	0.45
	≥60	65 (43.33)	13.00	
Gender	Male	111 (74.00)	12.00	0.396
	female	39 (26.00)	16.00	
Smoking	Somker	110 (73.33)	14.00	0.882
	Non-smoker	40 (26.67)	12.00	
Clinical phage	L D^1^	59 (39.33)	18.00	**<0.001**
	E D^2^	91 (60.67)	10.00	

L D^1^ means Limited Disease and E D^2^ means Extensive Disease.

Fresh serum and urine samples were obtained from 70 patients with SCLC from oncology department in Binzhou Medical University Hospital, China (48 males and 22 females with an average age of 52.3±11.3 year). A total of 45 patients had extensive disease defined as disease that has spread to the contralateral lung or that has distant metastases) and 25 patients had limited disease defined as disease that is confined to one lung, the mediastinum, and/or the regional lymph nodes. All patients voluntarily signed the informed consent. All patients were diagnosed with SCLC by pathological biopsy samples and had undergone chemotherapy and radiotherapy. A total of 40 healthy volunteers (20 males and 20 females with an average age of 38.7±12.1 years) were also studied. Healthy controls had no abnormal changes in blood, liver, heart, lung or kidney functions. This study was approved by the Medical Ethics Committee of Binzhou Medical University, China (Permit Number: BY2010008).

Human SCLC cell lines, including H1688, H446 and H345, were purchased from the Shanghai Cell Library of the Chinese Academy of Science. Cells were cultured in 1640 medium supplemented with 10% fetal bovine serum, 100 U/ml penicillin and 100 µg/ml streptomycin.

### Immunohistochemistry

The paraffin-embedded specimens were dewaxed in dimethylbenzene and hydrated in gradient alcohol. The sections were treated according to the following protocols: Endogenous peroxidase removed (incubation in 3% H_2_O_2_ for 30 min), antigen retrieved (about 95°C for 45 min), blocking with 10% goat serum (at room temperature for 30 min), primary antibody incubation overnight at 4°C, HRP-conjugated secondary antibodies incubation for 30 min at 37°C, DAB color reaction, hematoxylin staining, differentiation, dehydration and transparency. The dilution of primary antibodies was 1∶50 for ADAM-8, ADAM-10, ADAM-11 (Santa Cruz Biotechnology, Dallas, TX, USA), 1∶200 for ADAM-12 (Abgent, Suzhou, China), ADAM-15 and ADAM-17 (R&D Systems, Minneapolis, MN, USA). Two pathologists who were blinded to the clinical diagnosis performed the staining scores. In the event of a disputed result, consensus was reached by discussion. A semi-quantitative scoring system was used to grade the samples according to the percentage of positive staining: grade 0 (negative), grade 1 (weak positive, positive expression was less than 10%), grade 2 (moderate positive, positive expression was between 10% and 60%) and grade 3 (strong positive, positive expression was greater than 60%) [20].

### ELISA

Serum and the first urine of the day (mid-stream) were obtained from patients with SCLC, centrifuged (1000 g, 30 min) to collect the supernatant and stored at –80°C [21]. The serum and urine levels of ADAM-12 were measured using an ADAM-12 specific ELISA kit (R&D Systems). A volume of 50 µl of 2-fold diluted samples was added to a 96-well plate covered with ADAM-12 specific monoclonal antibody and incubated for 2 h at room temperature. After aspirating and washing each well four times, 200 µl of ADAM-12 conjugate was added and the wells incubated for 2 h at room temperature. Each well was washed an additional four times to remove residual liquid and 200 µl of substrate solution was added to each well and the wells incubated for 30 min in darkness. Stop buffer was added to terminate the reaction and the absorption intensity was read at 450 nm.

### Small interference RNA (siRNA) treatment

siRNAs for target genes were synthesized and modified (Invitrogen, Carlsbad, CA, USA). The sequence of the siRNA targeting ADAM-12 was 5′ GGA AGA GCU GAU GAA GUU GTT 3′ [22] and the scramble siRNA was 5′ UUC UCC GAA CGU GUC ACG UTT 3′. H1688 cells were cultured in 6-well plates and transfected with 75 pmol siRNA and 7.5 µl Lipofectamine 2000 (Invitrogen) was added to every well when the cell density was about 30%–50%. After 4 h, the Opti-media was replaced by normal media and the proteins were collected at 48 h after interference.

### Transfection of ADAM-12-L and ADAM-12-S in H345 cells

H345 cells were transiently transfected with ADAM-12-L and ADAM-12-S expression vectors (pcDNA3.1 plasmids containing human all length cDNA of ADAM-12-L or -S were gifts from Ulla M. Wewer in Copenhagen University) using Lipofectamine 2000 (Invitrogen). The cells transfected with ADAM-12-L were named H345-L and the cells transfected with ADAM-12-S were named H345-S.

### Real time PCR

Total RNA was extracted using a Trizol Kit (Takara, Dalian, China). cDNA was prepared using a cDNA synthesis kit (Thermo Scientific, Waltham, MA, USA). Real-time PCR was performed with SYBR Green (Thermo Scientific). The primers of the target genes were as follows:

F: 5′-GCAGTTTCACGGAAACCCAC-3′,

R: 5′-ACACGTGCTGAGACTGACTG-3′ for ADAM-12;

F: 5′-TCCTGTGTCTTCTTGCTGCC-3′,

R: 5′-GCGCACACACCTTAGTTTTTC-3′ for ADAM-12-L;

F: 5′-CCACCCATTCCATCTCCATC-3′

R: 5′-TTCTGCAAACCCTCAAACC-3′for ADAM-12-S

F: 5′-GAGCACAGAGCCTCGCCTTT-3′

R: 5′- CGCGGCGATATCATCATCCA-3′ for beta-actin.

### Western blotting

Cells were lysed with SDS lysis buffer containing a protease inhibitor mixture. The quantitative proteins were separated by SDS-PAGE electrophoresis and the proteins were transferred onto NC members. The member was blocked with 5% fat-free milk for 1 h with slight shaking and incubated with primary antibodies overnight at 4°C (1∶500 for ADAM-12, Abgent, Suzhou, China; 1:3000 for beta-actin, Cell Signaling Technology, Inc. Danvers, MA, USA), then incubated with HRP-conjugated secondary antibodies for 40 min at room temperature. Immunoblot bands were visualized using the ECL system (Millipore, Billerica, MA, USA) and X-ray film.

### Transwell experiments

Transwell experiments were conducted according to the manufacturer’s guide (BD, Franklin Lakes, NJ, USA). The Matrigel was diluted with media free serum (1:2). A volume of 60 µl of gel was plated into the upper chamber and solidified by incubating at 37°C for 3 h. 1×10^4^ cells were plated into the chamber, filtered into the lower chamber and introduced into the serum. Then cells were stained using Giemsa Dye, and the number of cells in 3 fields was counted under a light microscope.

### Cell cycle analysis

Cells were starved for 24 h by serum withdrawal. H1688 and H345 cells were treated. Cells were collected, fixed and incubated with RNase A (Thermo Scientific) for 30 min at 4°C. Then, cells were then incubated with Propidium iodide (Sigma-Aldrich, St. Louis, MO, USA) for 30 min. DNA content was detected via flow cytometry [23].

### Animal experiments

H1688 cells (a classical small cell lung cancer cell line) were cultured in 1640 medium and 10^6^ cells were administered subcutaneously into nude mice. The tumor size was monitored every week. When the tumor diameter reached approximately 1 cm, the mice were euthanized under anesthesia and tumors were excised. Excised tumors (called parental cells) were cut in half. One half was stored at –80°C, and the other was cut into pieces, digested (0.25% trypsin incubation for 15min) and filtered. Filtered cells were cultured again in normal medium. Three days later, cells were injected into immunodeficient mice via the tail vein. Ten weeks later, the mice were euthanized under anesthesia. Of the 10 mice that were injected, 5 mice had visible tumors (diameter>0.5 cm) in the abdomen lung and liver and the other 5 mice had no visible tumors. Compared with tumors resulting from subcutaneous injection, tumors resulting from the tail vein injection had higher levels of infiltration and invasion [24]. Thus, a highly metastatic animal model simulating tumor metastasis in patients was successfully constructed. The Medical Ethics Committee of Binzhou Medical University approved this study (Permit Number: BY2013001).

### Statistical analysis

All data were analyzed using SPSS 17.0 software. The Kaplan-Meier method and Log-Rank test were used to assess the univariate survival rate. Cox’s regression was used to perform the multivariate survival analysis. A receiver operating characteristics (ROC) curve analysis was performed to assess the cut-off for serum and urine levels of ADAM-12 in patients with SCLC and healthy volunteers. The area under curve (AUC) and p-values were evaluated. The expression differences between different groups were analyzed using paired *t* test. *P* < *0.05* was considered to be statistically significant.

## Results

### The expression of ADAMs in SCLC samples by IHC

**Figure 1:**
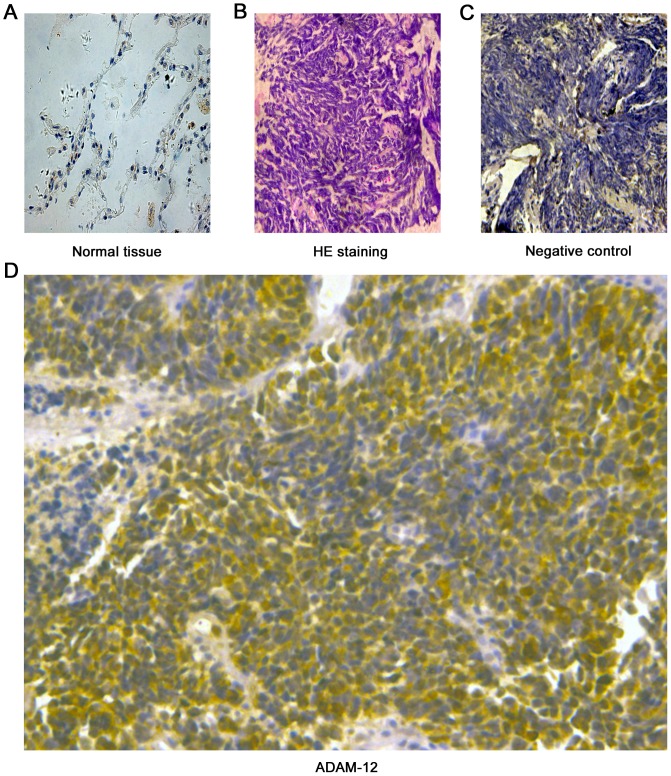
The expression of ADAM-12 in small cell lung cancer tissue samples. ADAM-12 was detected in small cell lung cancer tissue by immunohistochemistry staining. The dilution ratio of primary antibody was 1:200 for ADAM-12. (A) Normal lung tissue; (B) HE staining in SCLC tissue; (C) Negative control (incubated with PBS instead of primary antibody overnight at 4°C); (D) Staining for ADAM-12 in SCLC tissue.

The expression of ADAM-8, -10, -11, -12, -15 and -17 was evaluated by IHC in 150 SCLC samples. The level of positive expression (scores of grades 2 and 3) of ADAM-8, -10, -11, -15 and -17 was 31.33% (47/150), 34.66% (52/150), 19.33% (29/150), 39.33% (59/150) and 10% (15/150), respectively (**Figure S1**). The positive expression of ADAM-12 was the highest at 72.67% (109/150) and the strong positive expression (grade 3) of ADAM-12 was significantly greater compared with the other ADAMs (*P*<0.001). As shown in **Figure 1**, we found that ADAM-12 was expressed in SCLC and was located in the cytoplasm, but not the nucleus. HE staining indicated that the tumor tissue type had typical SCLC clinicopathological characteristics (larger nuclei and almost no cytoplasm). Tissue samples that were incubated overnight with PBS, instead of primary antibody, were used as negative controls. ADAM-12 was expressed significantly more in SCLC tissue samples compared with the expression of other ADAMs (*P*<0.001) indicating that ADAM-12 may play an important role in the process of proliferation, invasion and metastasis in SCLC.

### ADAM-12 is an independent prognostic factor in SCLC patients

**Figure 2:**
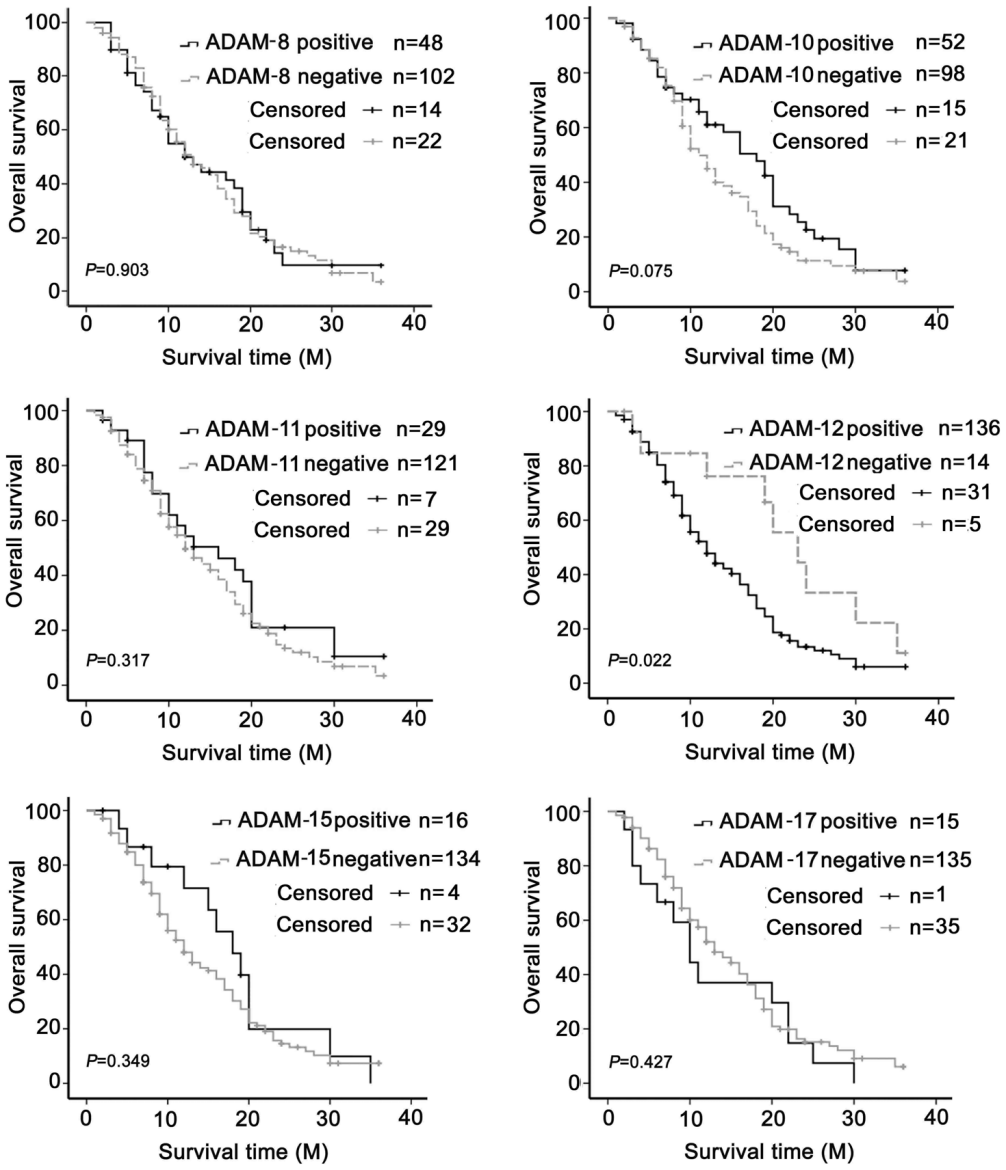
Survival curve by univariate survival analysis. Patient survival time was obtained by follow-up. Univariate survival analysis was performed to assess whether ADAM-8, -10, -11, -12, -15 and -17 could be considered as independent prognostic factors. The result showed ADAM-12 to be an independent and adverse factor (*P* = 0.022).

Since ADAM-12 was highly expressed in SCLC, we investigated the relationship between ADAM-12 and disease prognosis. All patients had accepted systemic therapy and 15 of 150 patients were still alive in January 2013 (closing date of the study). The average survival time of patients was 12.59 months and the 2-year survival rate was 16%. According to the IHC staining scores, those with grades 0 and 1 were considered to be in the negative group and those with grades 2 and 3 were considered to be in the positive group. Univariate survival analysis showed that the significant clinical factors that affected survival were extensive disease (*P*<0.001). Neither smoking (*P* = 0.882), gender (*P* = 0.396) was a significant prognostic factor nor were levels of ADAM-8 (*P* = 0.903), ADAM-10 (*P* = 0.075), ADAM-11(*P* = 0.317), ADAM-15 (*P* = 0.349) or ADAM-17 (*P* = 0.427). However, ADAM-12 (*P* = 0.022) was a significant negative prognostic factor (**Figure 2**). Multivariate survival analysis was performed to assess the prognostic influence of various factors. The results were consistent with the univariate survival analysis. Clinical stage (*P* = 0.001, HR = 2.003, 95% CI: 1.330 to 3.015) and level of ADAM-12 expression (*P* = 0.049, HR = 0.443, 95% CI: 0.197 to 0.995) were significant independent prognostic factors (**Table 2**) and ADAM-12 was closely associated with the clinical stage (*P*<0.001). Clinical characteristics of patients who were positive for ADAM-12 were compared to those who were negative for ADAM-12 in order to further clarify the role of ADAM-12 as an independent prognostic factor in SCLC. The results showed that the pathological stage was statistically significant (*P* = 0.037), while other factors including age (<65 compared to ≥ 65, *P* = 0.325), gender (*P* = 0.975), smoking (*P* = 0.743) and tumor size (*P* = 0.511) were not significant.

**Table 2:** Clinical and IHC variables influencing prognosis assessed by Multivariate survival analysis.

Parameter	Univariate survival analysis (*P* value)	Multivariate survival analysis (*P* value)	HR	95% CI
Gender	0.396	0.193	0.751	0.487–1.156
Smoking	0.882	0.941	0.983	0.662–1.554
Clinical stage	**<0.001**	**0.001**	2.003	1.330–3.015
ADAM-8	0.903	0.479	1.166	0.762–1.784
ADAM-10	0.075	0.208	1.319	0.857–2.030
ADAM-11	0.317	0.912	1.029	0.624–1.695
ADAM-12	**0.022**	**0.049**	0.443	0.197–0.995
ADAM-15	0.349	0.547	0.815	0.420–1.585
ADAM-17	0.427	0.659	0.876	0.485–1.581

### Serum and urine ADAM-12 levels in SCLC patients

**Figure 3:**
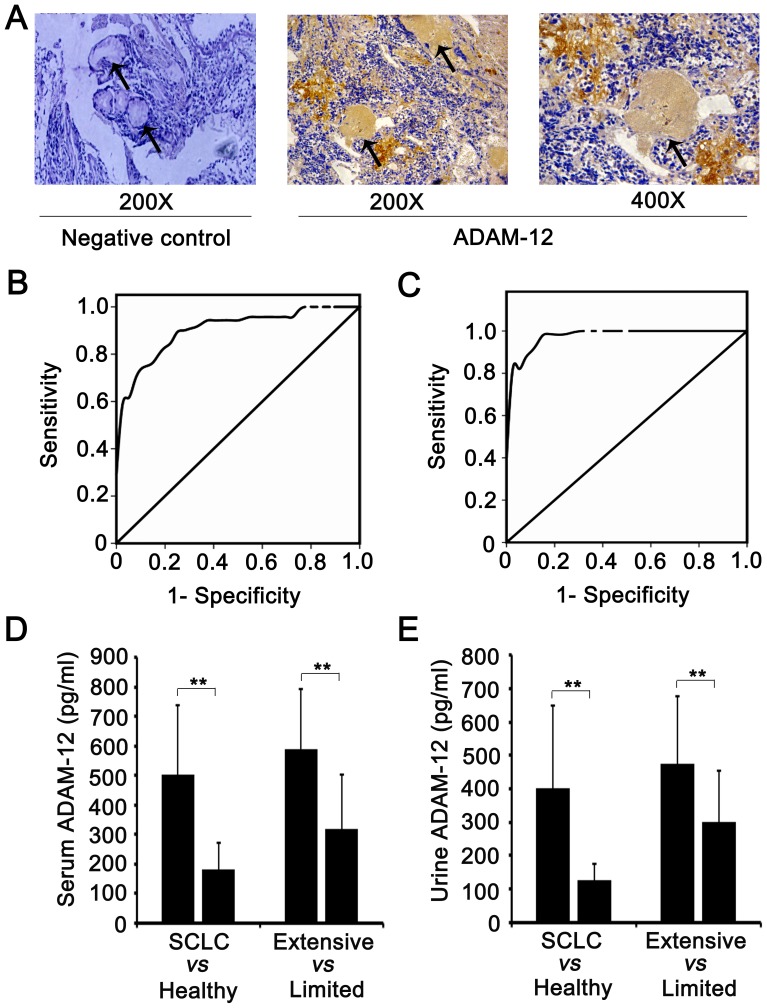
The expression of ADAM-12 in serum and urine. (A) ADAM-12 staining was strongly positive in mucus secretions (the region is indicated by the arrow). (B, C) An ROC curve was performed to assess the cut-off for patients with SCLC and healthy volunteers; ROC  =  receiver operating characteristics. (D, E) The expression levels of ADAM-12 in serum and urine from SCLC patients and normal controls were detected using an ELISA kit. ** *P*<0.01, SCLC patients *vs.* normal controls, SCLC patients with extensive disease *vs.* limited disease.

IHC staining demonstrated ADAM-12 to be highly expressed in mucus secretions, except for SCLC cells **(** **Figure 3A** **)**. We then inferred that ADAM-12 was likely secreted into the blood and excreted through the urine. Thus, we investigated the levels of ADAM-12 in the serum and urine of SCLC patients. Serum and urine from 70 SCLC patients and 40 healthy volunteers were collected, centrifuged and stored at –80°C. An Elisa kit was used to detect the expression of ADAM-12. The ROC curves yielded a higher AUC of 0.899 (*P*<0.001, 95% CI: 0.842 to 0.956) for 346 pg/ml in serum ADAM-12 level from SCLC patients (**Figure 3B**) than normal controls (a AUC of 0.847 for 105 pg/ml), and a higher AUC of 0.979 (*P*<0.001, 95% CI: 0.959 to 0.999) for 258 pg/ml in urine ADAM-12 level from SCLC patients (**Figure 3C**) than normal controls (a AUC of 0.869 for 92 pg/ml). The serum ADAM-12 level was significantly higher in SCLC patients than in healthy volunteers (502±234 *vs* 180±92 pg/ml, *P*<0.001) and in those with extensive disease compared to those with limited disease (586±205 *vs* 317±185 pg/ml) (**Figure 3D**). The urine ADAM 12 level was consistent with the serum level and was higher in SCLC patients than in healthy volunteers (404±247 *vs* 128±50 pg/ml) and in patients with extensive disease compared to patients with limited disease (477±201 *vs* 303±152 pg/ml) (**Figure 3E**). This indicates that ADAM-12 levels in serum and urine are correlated with SCLC progression and that ADAM-12 can be considered to be a diagnostic marker for SCLC disease since the expression of ADAM-12 increased with the development and progression of disease.

### ADAM-12 affected metastasis and proliferation in H1688 cells

**Figure 4:**
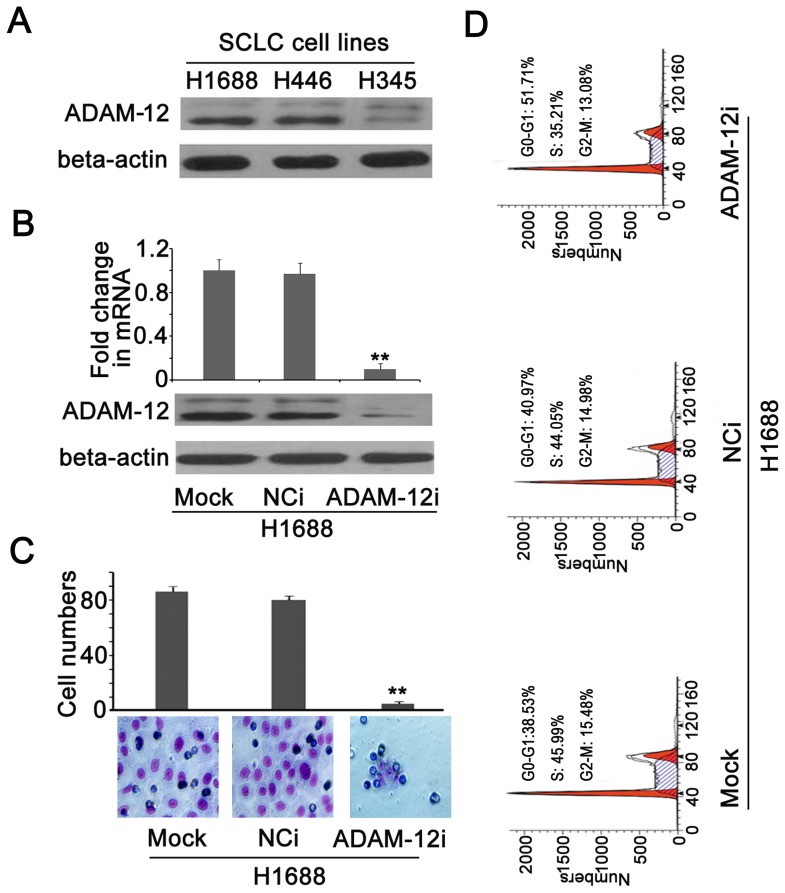
Analysis of proliferation and metastasis in H1688 cells after interfering with ADAM-12 expression. (A) The expression of ADAM-12 was higher in H1688 and H446 cells compared with H345 cells by Western blotting. (B) The mRNA and protein expression of ADAM-12 was significantly reduced when ADAM-12 expression was silenced by a specific siRNA targeting ADAM-12 (ADAM-12i) compared with untreated (Mock) and scrambled siRNA (NCi) in H1688 cells, ** *P*<0.01. (C) The cells induced by the serum filtering chamber membrane were significantly reduced in the ADAM-12i group compared with the Mock and NCi groups in H1688 cells, ** *P*<0.01. (D) The cell cycle was significantly arrested in the G0-G1 phase in the ADAM-12i group. Mock: untreated cells; NCi: the cells were treated with scrambled siRNA. ADAM-12i: the cells were treated with siRNA targeting ADAM-12.

As shown **Figs. 1** **,** **2** **and** **3**, ADAM-12 is an independent prognostic factor that might be useful as a diagnostic marker in SCLC. We subsequently explored the role of ADAM-12 in proliferation, invasion and metastasis in SCLC. First, we detected the expression of ADAM-12 in SCLC cell lines (H1688, H446 and H345) and found that ADAM-12 was more highly expressed in H1688 and H446 cells compared with the H345 cell line (**Figure 4A**). In a study on breast cancer, ADAM-12 was reported to have two transcription forms (membrane-anchored ADAM-12-L and secreted type ADAM-12-S) that play different roles [22]. Because of the sequence similarity, different siRNAs that targeted ADAM-12-L and ADAM-12-S, respectively could not be designed so siRNA was used to target ADAM-12. The expression of ADAM-12 was significantly decreased in the specific siRNA group compared with the untreated and the scrambled siRNA groups (**Figure 4B**). When there was interference to the expression of ADAM-12 in H1688 cells, the cells induced by serum filtering the chamber membrane were significantly reduced (**Figure 4C**) and the cell cycle was arrested in the G0-G1 phase (**Figure 4D**). Roy *et al* reported that ADAM-12-L promoted cellular proliferation and that ADAM-12-S promoted cellular migration in breast cancer [22]. In our research, cellular proliferation and migration were significantly inhibited after silencing the expression of ADAM-12 by specific siRNA in the H1688 cell line, demonstrating that ADAM-12 is associated with cellular proliferation, invasion and metastasis. To clarify the roles of ADAM-12-L and -S in cellular proliferation and migration, an experiment was designed and carried out that is detailed below.

### ADAM-12-L promotes proliferation and ADAM-12-S promotes invasion

**Figure 5:**
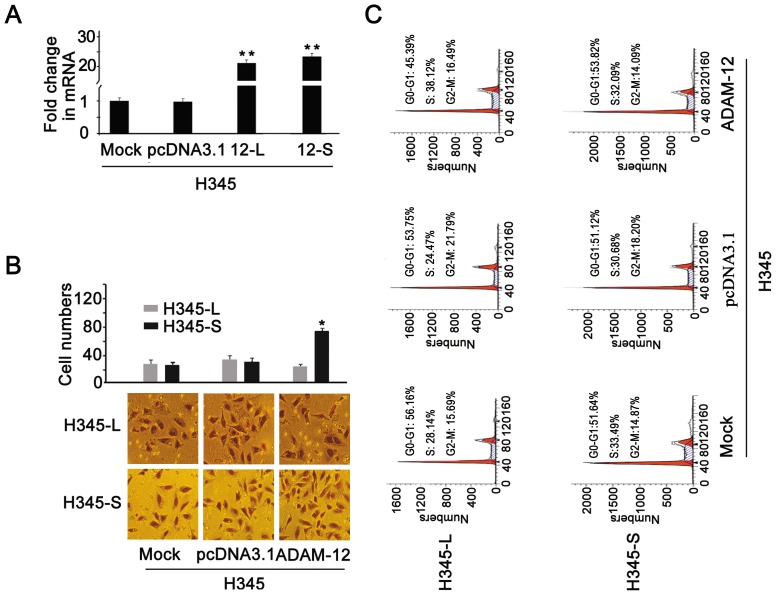
The functional analysis of ADAM-12-L and -S in H345 cells. (A) The mRNA expressions of ADAM-12-L and -S were detected by real time PCR in H345 cells transfected with the expression vector of ADAM-12-L and -S, ** *P*<0.01. Mock: untreated group; pcDNA3.1: negative group. 12-L: transfected with pcDNA3.1-ADAM-12-L; 12-S: transfected with pcDNA3.1-ADAM-12-S. (B) The cells induced by the filtering chamber membrane were not significantly different in the H345 cells transfected with pcDNA3.1-ADAM-12-L (named H345-L), however the cells were significantly increased in H345 cells transfected with pcDNA3.1-ADAM-12-S (named H345-S), * *P*<0.05. (C) ADAM-12-L induced cells to go from the G0-G1 phase into the S phase, but ADAM-12-S did not influence cellular proliferation in H345 cells.

As shown **Figure 4**, when the ADAM-12 expression was silenced, cellular proliferation and migration was significantly inhibited in H1688 cells. ADAM-12 expression was lower in H345 cells compared with H1688 and H446 cells (**Figure 3A**). H345 cells were then transiently transfected with the expression vector of ADAM-12-L (H345-L) or -S (H345-S). The transfection efficiency was assessed by real-time PCR since there was no specific antibody targeting ADAM-12-L or -S. The mRNA expression of ADAM-12-L and -S were significantly increased after transfection (**Figure 5A**). There was no significant difference between H345-L cells induced by filtering through the chamber membrane and mock (normal control) or negative controls (pcDNA3.1). However, the contradictory results were found in H345-S cells. After transfecting with ADAM-12-S, H345 cells induced by serum filtering through the chamber membrane were significantly increased, indicating that ADAM-12-S, not ADAM-12-L, plays an important role in SCLC invasion and metastasis (**Figure 5B**). Meanwhile, cell cycle analysis showed that H345-L cells entered into S phases, but that H345-S cells were not significantly different compared with negative control cells (**Figure 5C**). This indicated that ADAM-12-L could promote cell proliferation and ADAM-12-S could promote cell invasion and metastasis, which is consistent with Roopali’s result [22].

### The expression of ADAM-12 was increased when tumor invasion and metastasis was enhanced

**Figure 6:**
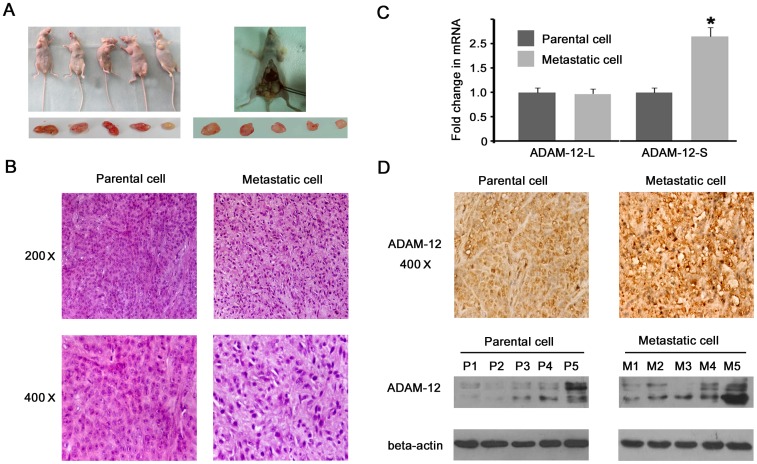
ADAM-12 was up-regulated when the invasion and metastasis of SCLC cells was enhanced. (A) A highly metastatic animal model was constructed, 1×10^6^ cells were administered subcutaneously and the subcutaneous tumors (parental cells) were injected into nude mice via tail vein to form metastatic tumors (metastatic cells). (B) Morphological differences were observed between parental cells and metastatic cells via hematoxylin and eosin (HE) staining. Compared with parental cells, metastatic cells were smaller, had shoaled cytoplasm staining, deeper nuclear staining, thinner cell density and weaker cell compact. (C) There was no difference in ADAM-12-L mRNA expression between parental and metastatic cells, however ADAM-12-S mRNA was significantly higher in metastatic cells, * *P*<0.05. (D) ADAM-12 protein expression was significantly increased in metastatic cells by IHC and western blotting. P1-P5 indicates the five subcutaneous tumors and M1-M5 indicates the five metastatic tumors.

Previous research [22] had reported that ADAM-12-L promoted cell proliferation and ADAM-12-S promoted cell invasion and metastasis in an animal model; we confirmed this conclusion on a cellular level. However, there have been no reports on whether the expression of ADAM-12 is increased when tumor invasion and metastasis are enhanced. As shown in **Figure 3**, serum and urine ADAM-12 levels were significantly increased in patients with extensive disease when compared to those with limited disease, suggesting that the expression of ADAM-12 is increased with the development of disease. In order to confirm this conclusion, the highly metastatic animal model simulating tumor metastasis was constructed as described in the Methods (**Figure 6A**). There was a greater change in the morphology of the metastatic cells compared with the parental cells from this model. Metastatic cell size was smaller, the cytoplasm stain was shoaled, the nuclear stain was deepened, the cell density thinned and the cell compact weakened (**Figure 6B**). The expression of ADAM-12 was detected in parental and metastatic cells. The mRNA expression of ADAM-12-L was no different between parental and metastatic cells, but ADAM-12-S mRNA was significantly higher in metastatic cells (**Figure 6C**). This indicates that ADAM-12-S, not ADAM-12-L, plays an important role in invasion and metastasis. The ADAM-12 protein was detected by IHC and Western blot. The result showed that ADAM-12 was obviously higher in metastatic cells compared with parental cells (**Figure 6D**).

## Discussion

There are more than 30 ADAMs that belong to the metalloproteinase family. They interact with integrins to mediate cell-cell and cell-extracellular matrix interactions [25], shed a large number of transmembrane proteins, degrade the ECM and activate the Notch and EGFR pathways to promote cell proliferation, invasion and metastasis [6].

The main features of SCLC are rapid growth and extensive metastasis in the early stage. At present, there is no effective diagnostic marker to assist in the diagnosis of SCLC, especially during the early stage. This study was designed to assess the expression of ADAMs in SCLC and in order to find a useful diagnostic marker. Until now, only the expression of ADAM-15 had been detected and the expression of other ADAMs had been only seldomly reported in SCLC [8]. In our research, we detected ADAM-8, -10, -11, -12 and -17 and found that ADAM-12 was more highly expressed compared with other ADAMs in SCLC clinical samples.

ADAM-12 is highly expressed in numerous cancers including breast cancer [12], [14], [26], liver cancer [16], stomach cancer [26], [27], colon cancer [26] and nervous system cancer [28]. In present study, univariate and multivariate survival analysis indicated that ADAM-12 is likely an independent prognostic factor indicates poorer survival in SCLC patients. Although ADAM-12 is overexpressed in numerous tumors, ADAM-12 as a specific diagnostic marker has only been used for breast cancer [22]. In our study, we found that the ADAM-12 level was significantly increased in SCLC patients compared with normal controls and in patients with extensive disease compared to those with limited disease, suggesting that ADAM-12 might be as an effective diagnostic and prognostic marker.

ADAM-12 is a complex, multi-domain protein that regulates cell proliferation and movement. It can shed heparin-binding, epidermal growth factor-like growth factor (HB-EGFR) to activate the EGFR signal pathway [29], [30], shed delta-like 1 to activate the Notch signal pathway [31], interact with the type II receptor to activate the TGF-beta signal pathway [32], interact with β1-integrin to regulate cell migration [33], and can promote angiogenesis [34]. Since ADAM-12 was found to be highly expressed in SCLC patients, the function of ADAM-12 in promoting cell proliferation, invasion and metastasis was explored. In non-small cell lung cancer, only ADAM-12-L and not ADAM-12-S was expressed in tissue samples and cell lines. The overexpression of ADAM-12-L was associated with the poorer differentiation, a higher relapse rate and a poorer prognosis [35]. In SCLC tissue samples and cell lines, ADAM-12-L and ADAM-12-S were both present. When the expression of ADAM-12 was silenced in H1688 cells, cell proliferation and invasiveness was significantly inhibited. When ADAM-12-L or ADAM-12-S was supplemented in H345 cells, ADAM-12-L promoted cell proliferation and ADAM-12-S promoted cell invasiveness, which was consistent with the observations reported in breast cancer [22]. The highly metastatic animal model confirmed that the expression of ADAM-12 was increased when tumor invasion and metastasis was enhanced, suggesting that ADAM-12 plays an important role in SCLC progression, invasion and metastasis.

In conclusion, this is the first study, to our knowledge, that demonstrates the expression of ADAMs in SCLC patients and confirms that ADAM-12 is an independent prognostic factor and an effective diagnostic marker that is involved in SCLC proliferation, invasion and metastasis.

## Supporting Information

Figure S1 **The expression of other ADAMs in SCLC by IHC.** ADAM-8, -10, -11, -15 and -17 were detected in small cell lung cancer tissue by IHC staining. The dilution ratio of primary antibody was 1:50 for ADAM-8, -10, -11 and 1:200 for ADAM-15 and -17.
(DOCX)
