# Supplementary figures and images for: ADAM-12 as a Diagnostic Marker for the Proliferation, Migration and Invasion in Patients with Small Cell Lung Cancer
Source: PLoS One. 2014 Jan 21;9(1):e85936. doi: 10.1371/journal.pone.0085936 (PMC3897605; doi:10.1371/journal.pone.0085936)

Figure S1. The expression of other ADAMs in SCLC by IHC.


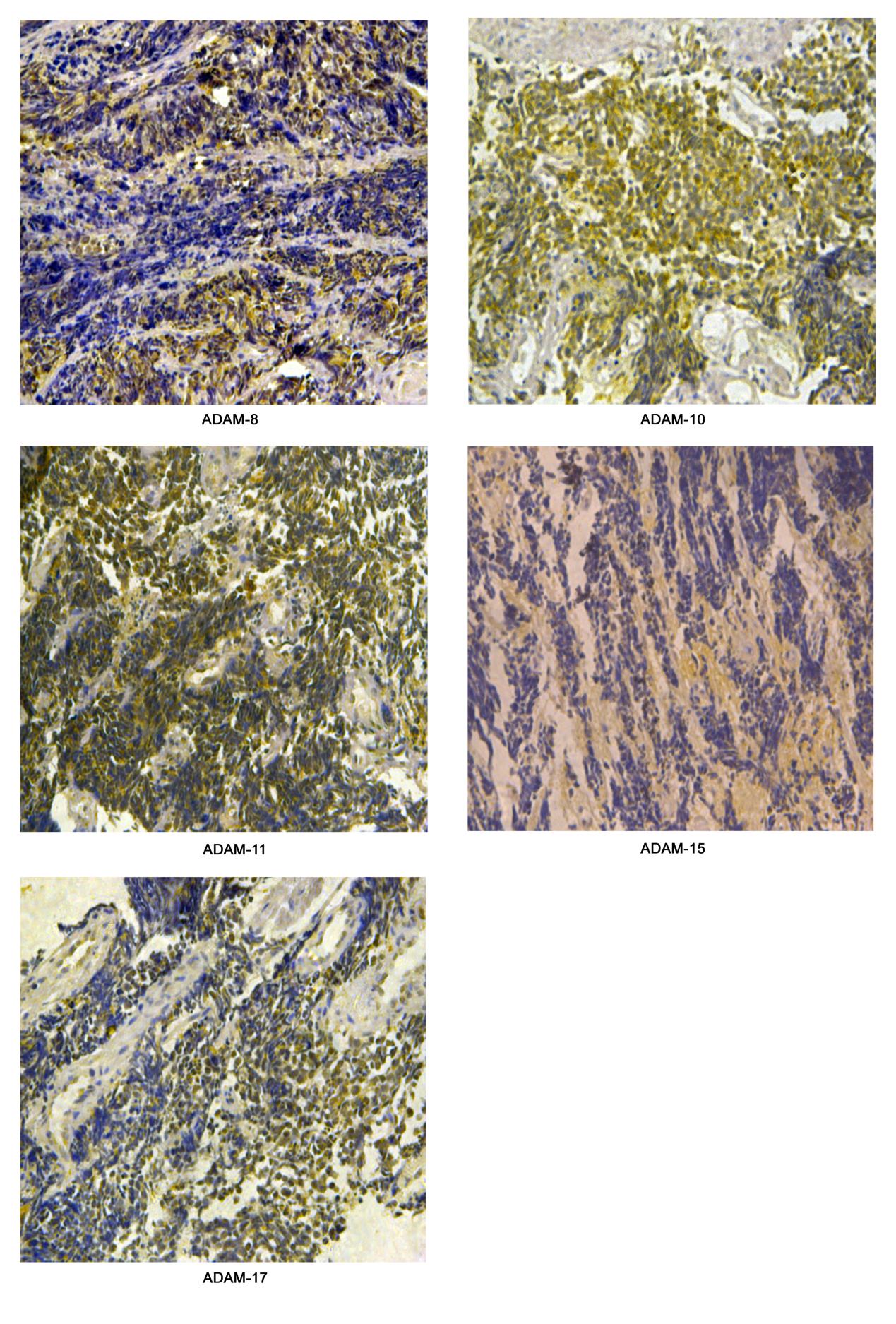

Supplement: Figure S1 — The expression of other ADAMs in SCLC by IHC. ADAM-8, -10, -11, -15 and -17 were detected in small cell lung cancer tissue by IHC staining. The dilution ratio of primary antibody was 1:50 for ADAM-8, -10, -11 and 1:200 for ADAM-15 and -17. (DOCX) [file pone.0085936.s001.docx]
